# Supplementary material for: Causal Relationship between Aging and Anorexia Nervosa: A White-Matter-Microstructure-Mediated Mendelian Randomization Analysis
Source: Biomedicines. 2024 Aug 16;12(8):1874. doi: 10.3390/biomedicines12081874 (PMC11351342; doi:10.3390/biomedicines12081874)
Supplement: Supplementary file 1 [file biomedicines-12-01874-s001.zip › Supplementary Figure/Figure S4/Singe SNP plot of MR analysis for TL to ubm-b-1980.pdf]

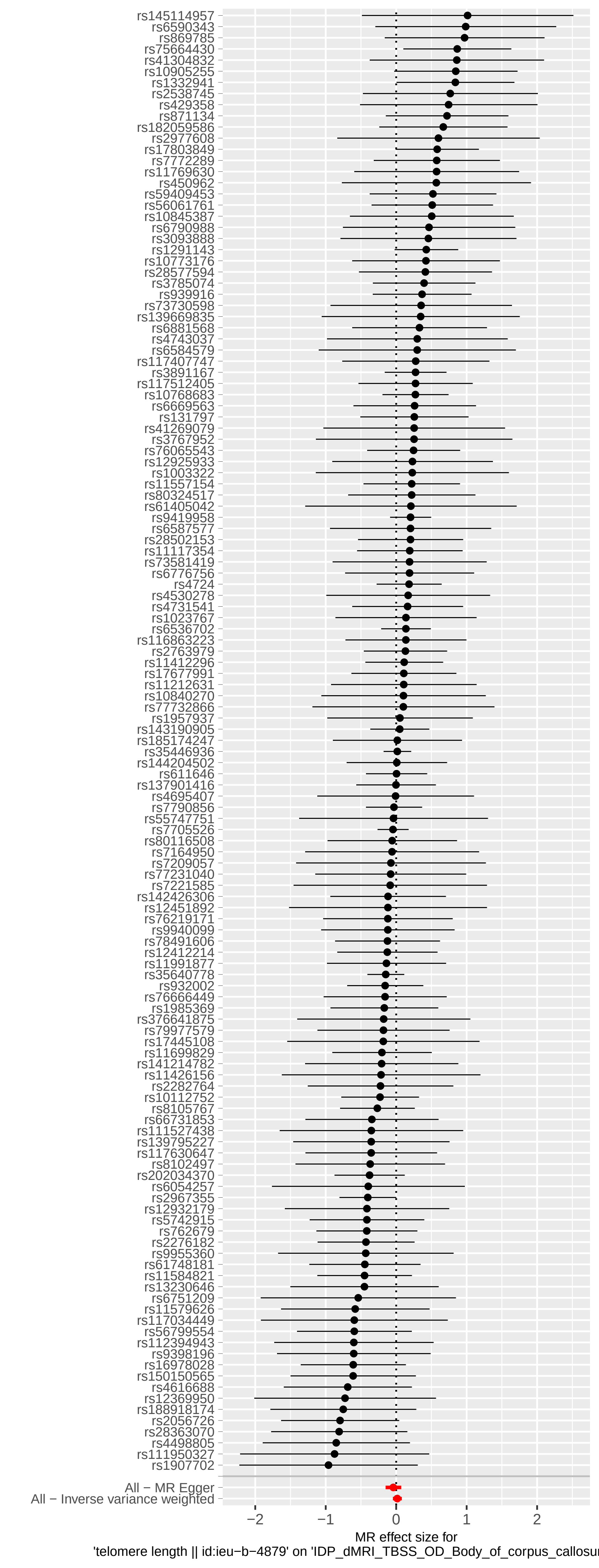
rs145114957
rs6590343
rs869785
rs75664430
rs41304832
rs10905255
rs1332941
rs2538745
rs429358
rs871134
rs182059586
rs2977608
rs17803849
rs7772289
rs11769630
rs450962
rs59409453
rs56061761
rs10845387
rs6790988
rs3093888
rs1291143
rs10773176
rs28577594
rs3785074
rs939916
rs73730598
rs139669835
rs6881568
rs4743037
rs6584579
rs117407747
rs3891167
rs117512405
rs10768683
rs6669563
rs131797
rs41269079
rs3767952
rs76065543
rs12925933
rs1003322
rs11557154
rs80324517
rs61405042
rs9419958
rs6587577
rs28502153
rs11117354
rs73581419
rs6776756
rs4724
rs4530278
rs4731541
rs1023767
rs6536702
rs116863223
rs2763979
rs11412296
rs17677991
rs11212631
rs10840270
rs77732866
rs1957937
rs143190905
rs185174247
rs35446936
rs144204502
rs611646
rs137901416
rs4695407
rs7790856
rs55747751
rs7705526
rs80116508
rs7164950
rs7209057
rs77231040
rs7221585
rs142426306
rs12451892
rs76219171
rs9940099
rs78491606
rs12412214
rs11991877
rs35640778
rs932002
rs76666449
rs1985369
rs376641875
rs79977579
rs17445108
rs11699829
rs141214782
rs11426156
rs2282764
rs10112752
rs8105767
rs66731853
rs111527438
rs139795227
rs117630647
rs8102497
rs202034370
rs6054257
rs2967355
rs12932179
rs5742915
rs762679
rs2276182
rs9955360
rs61748181
rs11584821
rs13230646
rs6751209
rs11579626
rs117034449
rs56799554
rs112394943
rs9398196
rs16978028
rs150150565
rs4616688
rs12369950
rs188918174
rs2056726
rs28363070
rs4498805
rs111950327
rs1907702
All − MR Egger
All − Inverse variance weighted
−2
−1
0
1
2
MR effect size for
'telomere length || id:ieu−b−4879' on 'IDP_dMRI_TBSS_OD_Body_of_corpus_callosu
